# Leveraging 5G network for digital innovation in small and medium enterprises: a conceptual review

**DOI:** 10.1186/s13731-021-00181-5

**Published:** 2022-05-26

**Authors:** Maxwell Olokundun, Mercy Ejovwokeoghene Ogbari, Hezekiah Falola, Ayodotun Stephen Ibidunni

**Affiliations:** Department of Business Management, Covenant University, Ota, Ogun State Nigeria

**Keywords:** SMEs, 5G mobile technology, Digital innovation

## Abstract

Small- and medium-sized enterprises (SMEs) play a crucial role within a country’s economy considering that they provide a source of skills and innovation for entrepreneurship and their capacity for introducing, integrating and disseminating new technologies is incomparable. However, the world may be looking at an impending devastating recession delivered by the coronavirus pandemic. As governments intensify restrictions on business to halt the spread of the pandemic, the fear of the virus also reconfigures the very concept of business and the workplace. Therefore, there is a need for a greater focus on digital transformation considering that physical distancing requirements to curb the spread of this virus may become a cultural orientation for businesses and the workplace. A real digital transition is anticipated for small businesses with the 5G network technology. 5G network will not just be a technological connection but will affect different socio-economic sectors and will stimulate innovation in small companies. The goal of this paper was to carry out a literature review on the potential ways small businesses can leverage on 5G network for digital innovation. The paper proposed that small and medium businesses can leverage the 5G Mobile Technology through enhanced interpersonal communication, expanded remote work opportunities, innovative digital tools and supply chain efficiency. Finally, while many individuals and small business owners will profit from 5G technology some will work to undermine it. As a result, companies must keep this in mind when implementing 5G, or other new technology, and adjust their security protocols accordingly to remain secure.

## Introduction

Small businesses can expect a true digital transition with 5G (fifth generation of broadband data-networking digital communications technology) network technology (Tullberg et al., 2016). With an estimated annual growth rate of 97% and approximately 40% of the global population using IoT apps on a 5G network, the new mobile technology has the potential to generate a $251 trillion economic impact by 2025. This growth can be attributed to the fact that the 5G network will not only provide a technological connection but will also have an impact on various socioeconomic sectors and encourage small business innovation (Webb, 2016). The new technological link will make it possible to implement well-known initiatives, such as Smart City, Smart Company, and artificial intelligence in business (Zhu et al., 2016). Because of increased data transfer speed and reduced time between signal transmission and reception, the 5G network will redefine the role of connectivity and connections in global society, providing a new foundation for business innovation, particularly in small businesses. The goal of this paper is to conduct a review of the literature on the various ways small businesses can use the 5G network for digital innovation.

## Methodology

This is a model paper aimed at developing a theoretical framework for predicting relationships between the implementation of 5G technology and digital innovation in SMEs. The proposed conceptual arguments will describe and explain the process by revealing antecedents, outcomes, and contingencies associated with the focal construct (5G Technology and Business Innovation). This paper will typically involve some theorising in which an attempt will be made to construct a network around the focal construct (5G Technology and Digital Innovation in SMEs) by employing a formal analytical approach to examine and detail the causal linkages and mechanisms at work. The goal would be to look into previously unexplored links between the focal construct (5G and Digital Innovation in SMEs). The following is the methodology.

Rationalism is an epistemological position in which reason is regarded as the primary source and test of knowledge, or any viewpoint in which reason is invoked as a source of knowledge or justification (Ormerod, 2009). As a result, the philosophy of rationalism is well-suited to this study, which heavily relies on reason as a measure of comprehension. Rationalism is a methodological approach in which the criterion of validity is intellectual and deductive rather than sensory. To that end, the researchers developed a set of prepositions at the start of the study to test the validity of the statements. The researchers employed an analytical research strategy that drew on previously available facts and data. To explain the ideology advanced in relation to the subject matter, the researchers studied and analysed available data and information in the literature. Using this strategy, the researchers searched the literature for critical details and new information to incorporate new ideas into the information being produced. A literature review was used as a research method. According to Onwuegbuzie et al. (2012), conducting a literature review is the same as conducting research, with the information gathered by the reviewer representing the data. An integrative review was conducted to analyse and synthesise the information gathered. The integrative review was used to provide a synthesis of knowledge as well as the study’s findings’ applicability to research and practise. Brown (2006) identifies five criteria for determining the validity of a literature review: intent/purpose, scope, authority, audience, and format. As a result, during the literature review, each of these requirements was considered. Furthermore, the most widely cited academic databases, such as Scopus, Web of Science, and Google/Google scholar, were used for online searches. The dependability of the review process was determined by analysing information with transparency and objectivity while evaluating evidence in the literature (Haddaway et al., 2015). Because no primary data was collected, no formal ethical approval was required for this study. However, ethical standards were upheld by acknowledging the source of information, acknowledging the ideas, theories, and conceptualizations of others, and being critical of the studies included in the review.

Therefore, this paper will interrogate the following prepositions.

1. 5G Technology will foster development of innovation in communication technologies.
2. 5G Technology will facilitate innovations in remote work opportunities.
3. 5G Technology will enhance innovations in Supply Chains.
4. 5G Technology will actuate the development of innovations in Digital Marketplaces.

## Conceptual framework

### Small and medium enterprises

Small- and medium-sized businesses (SMEs) or small- and medium-sized businesses (SMBs) have fewer than a certain number of employees. Global organisations, such as the World Bank, the EU, the United Nations, and the World Trade Organization (WTO), use the abbreviation "small and medium-sized business" (Kowo et al., 2019). Each country defines small- and medium-sized businesses differently. The country in which the organisation operates determines the size requirements of a SME (European Commission, 2018). Several factors influence a company's size or classification as a SME, depending on the country. Such considerations include annual revenue, employee count, business assets, or a combination of these factors (Lemmers, 2014). Most SMEs in the world are self-contained enterprises with no fewer than 50 employees. In developing countries, smaller (micro) and informal businesses outnumber larger (formal) businesses. Small- and medium-sized businesses rely heavily on innovation in many economic sectors (Buavait et al., 2019).

### Digital innovation

The application of emerging technology to current business issues or processes is referred to as digital innovation. The application of digital technologies to improve services, traditional business models, products, and processes is also known as "digital innovation" (Karimi & Walter, 2015). Smarter devices, better data storage and retrieval, and broader information dissemination are the outcomes of digital innovation (White, 2017). Businesses benefit from digital innovation in terms of operational efficiency, quality, and productivity (OECD, 2015). Innovation is driven by technology, and innovation is the path to business success. It's difficult to imagine a company that hasn't benefited from the digital revolution (Muller et al., 2016). Unquestionably, technology-driven innovation is today's recipe for business success. Because technology enables business innovation, a business cannot grow without a technological foundation (Obi et al., 2018).

### 5G technology

5G refers to the fifth generation of broadband data-networking digital communications technology. It is essentially an upgrade to the more popular 4G (fourth generation of broadband data-networking digital communications technology) and aims to improve connectivity not only between individuals but also between computers, objects, and apps (5G–PPP, 2016). Generation is represented by the letter "G," which is commonly associated with cellular networks. 5G is expected to significantly increase mobile network capacity over 4G and previous iterations, allowing more users to connect to the network than ever before (Kliks et al., 2018). The new 4G standard is 500 times faster than 3G (the third generation of broadband data-networking digital communications technology) and enables more sophisticated activities, such as video sharing and video conferencing (Lemstra, 2018). The most significant difference between 5G and previous iterations is the increased efficiency and decreased latency, which is the time between a device transmitting information and a receiver accessing it (Rendón Schneir et al., 2018).

## Synthesis with concepts

### Role of SMEs in an economy

In many countries, small- and medium-sized businesses account for more than 99% of all businesses (SMEs). They generate significant value-added investments and employment while also benefiting local economies (Iorun, 2014). Small- and medium-sized enterprises (SMEs), which have much lower investment costs than large corporations, help to alleviate poverty by creating long-term job opportunities in local communities and raising living standards (Osuagu, 2016). Small- and medium-sized businesses create more jobs than larger corporations. In a market economy, the private sector, particularly small- and medium-sized enterprises (SMEs), provides more long-term employment opportunities for transition economies (Wymenga, et al., 2013). Small- and medium-sized business assistance can assist large enterprises in reorganising by streamlining manufacturing processes as separate sold-off units with no direct connection to the primary operation (USITC, 2014). By providing complementary services, they manage the volatility of a global economy while controlling the hegemony of big business. Because of their adaptability and creativity, they raise the level of skills through inter-enterprise collaboration. As a result, SMEs should benefit significantly from developing skilled labour industries and expanding a well-equipped service sector capable of increasing GDP (Eniola, 2013). In general, a growing SME sector can increase societal and economic flexibility while also encouraging business innovation.

### Innovation and growth of SMEs

Innovation refers to the process of creating a new or improved product. Business innovation includes the development of new processes, procedures, or products that can result in significant improvements in business (Walobwa et al., 2013). In the end, a company will be revitalised, new value will be created, and growth and productivity will be enhanced. To thrive, a company must constantly innovate and develop. Opening new sales channels, leveraging existing networks, and ultimately generating higher profits are all part of successful business innovation. This should give businesses a competitive advantage. Technology has become a key driver of business strategy and innovation in almost every industry. Organizations must find ways to effectively innovate while utilising and improving existing tools and best practises (Muller, et al., 2015). In today's modern world, success necessitates innovative, forward-thinking approaches that embrace technology while also addressing the need to change traditional business practises.

### Digital innovation and SMEs

Digital innovation contributes to the creation of tools that businesses can use to solve complex problems. Data collection and analysis for scalability are made possible by improved hardware and smarter software (Andrews et al., 2018). This can be accomplished by having teams participate in video conferences, gauging public opinion on social media and business forums, and soliciting customer feedback via online surveys (Ezell, 2016). Digital innovation has transformed marketing; using online advertisement strategies is far more effective than traditional ads in identifying target audiences, discovering their needs, and developing a marketing plan to persuade them to buy (Hossain & Lassen, 2017). Customer service has improved because of digital innovation in a variety of channels, such as phone calls, emails, social media sites, webinars, and similar sites (Collin et al., 2015). Technology is becoming increasingly important in industry as the world of business moves towards it, making the distinction between the two nearly impossible (Internet Retailing, 2015). Because digital innovation creates businesses and technology paves the way, continued use of technology is required for innovation (Moreira et al., 2018).

## Results and discussion

### Preposition 1: 5G technology will foster development of innovation in communication technologies

Clear and effective communication is critical for fostering a community that encourages business innovation. This is consistent with the assertion of Park et al. (2015) that communication, including active listening, is more important than ever in our modern age, and that with the rate of change increasing rapidly, technological progress and the transformation of entire industries with unpredictable business models are possible (Schallmo et al., 2018). Simply put, most people prefer to respond to questions rather than understand them. This also supports the viewpoint of Schneir et al. (2018), who argue that for digitization to thrive and develop collective strategies with start-ups, investors, and clients all over the world, active ecosystem listening is required. With the introduction of a 5G mobile network, subscribers and downstream providers would have access to devices capable of transmitting and receiving data in real time. Hitch-free calls, as well as higher quality video meetings and access to non-latent chat apps, will become the norm (Lemstra, 2018). This is supported by Kliks et al. (2018), who demonstrate that using a wireless router (Wi-Fi), networking-related activities, such as internet transfers and cloud-based file sharing, can become simpler and more efficient, allowing businesses to increase productivity.

### Preposition 2: 5G technology will facilitate innovations in remote work opportunities

Remote work is a work style that allows professionals to work outside of the confines of a traditional business setting (Bloom, 2014). According to Friedman (2014), remotely working employees or company owners can conduct tasks and achieve their goals from anywhere rather than commuting to an office every day from a specific desk. Business innovation does not take place only in the boardroom, where one or two voices dominate conversations and the others are sometimes deafeningly silent. Grant et al. (2013) bolstered this notion, arguing that allowing remote working communicates to employees and teams the trust and confidence that has been placed in them to do their jobs efficiently. Remote work has become a collaborative practise for many businesses, but it presents unique challenges to the use of remote working techniques by organisations (Groysberg & Abrahams, 2014). Most businesses struggle with maintaining team cohesion and addressing challenges for separated team members when working with remote teams. While remote work provides companies with agility, better scheduling, and lower staff turnover, it can also make it difficult for teams to communicate, collaborate, and interact, according to Harker and MacDonnell (2012). As a result of the introduction of 5G, even more work tasks can now be completed remotely. This is due to the strength of connectivity, high speed, and frequency of data transfers, which will allow a variety of highly skilled workers to function from any venue or workplace, resulting in an increase in remote work opportunities (Kortis, 2017).

### Preposition 3: 5G technology will enhance innovations in Supply Chains

According to Bódogh (2014), the Google Cloud Platform and Digital Marketplaces are thriving today because of artificial intelligence and machine learning innovation. Similarly, Alhlou et al. (2016) stated that digital markets are online outlets for the sale of a variety of goods that can sell a variety of items or specialise in a product category. These technologies are used to create dependable virtual trading platforms and ecosystems that buyers and sellers rely on in their digital markets daily for a variety of tasks. As a result, according to Brave Software (2018), mobile and digital tools play an important role in business innovation, and effective businesses use technology to create digital workplaces to improve company cohesion. With the advent of smartphones, digital services, and marketplaces, such as Apple Store, Google Store, and other digital distribution channels, changed almost overnight (Clifton, 2015). These innovations were based on the development of the technology platform first, followed by an examination of the use cases. In contrast, 5G already has a potentially infinite number of use and interaction cases. 5G has the potential to catalyse, encourage, and create a slew of new IoT business cases, and businesses and the industry must be able to capitalise on that commitment (Damian, 2014). 5G would promote the creation of digital networks or marketplaces that connect consumers with providers of goods and services, thereby simplifying business. 5G will also increase the availability of infrastructure platforms that support electronic marketplaces and encourage digital business models (Heggde & Shainesh, 2018).

### Preposition 4: 5G technology will actuate the development of innovations in digital marketplaces

Supply chain innovation, according to Kern and Wolff (2019), is defined as improvements to the operation of supply chains in general, and specifically, the flow of products, information, work, and funds across supply chain systems. The supply chain, according to Ageron et al. (2013), is the backbone of a company and is versatile in supporting so many components of a business. However, supply chains are vulnerable in a variety of ways due to unneeded change, strain, and failure, with far-reaching consequences throughout the enterprise (Abdelkafi & Pero, 2018). The 5G network infrastructure promises shorter latency times and greater coverage, increasing the use of smart devices and, as a result, operational efficiencies and business digitisation (APICS, 2018.). 5G will pave the way for low-cost sensors to track a product from the manufacturing line to the warehouse and finally to the customer (Arlbjrn & Paulraj, 2013). The position and condition of the goods can be monitored in real time, giving businesses a clear picture of their stock, supply chain, and sales, as well as mitigating risks. These data will be analysed to provide incredible insights into how to further streamline operations (Arnold et al., 2016). Delivery vehicles will be able to respond to real-time data from a 5G network much more quickly than they can now, making deliveries safer and more efficient (Blossey et al., 2019). Smart transport systems will be able to operate in a secure and efficient port environment with higher bandwidth connections (Büyüközkan & Göçer, 2018). Warehouses must scale up and meet smart, efficient, and automated warehouse standards as e-commerce grows, due to increased demand for quick responses and the need to manage a greater number of stocks keeping units (SKUs) with fewer errors. Over the next decade, the preference for digital transformation is expected to rise, resulting in fundamental changes to warehouse operations (Crupi et al., 2020). As a result, the adoption of warehouse interruptive digitalization will reach a tipping point (Schmidt et al., 2015). Businesses that have already digitised their supply chains will have a better chance of benefiting from 5G technology (Stevens & Johnson, 2016).

## Implications and contributions to research and practice

This paper makes a significant contribution to research and practise. In terms of research, the theoretical arguments presented in this paper advance a framework based on the notion that 5G technology holds promise for business innovation, especially for small businesses. Our study, as a theoretical paper, raises several avenues for future research, both in terms of theory development and concept validation. More research is needed to fine-tune and expand on the proposed framework. To begin, while we have generated a few useful conceptual linkages with an emphasis on the proclivity of 5G technology to foster digital innovation, particularly among SMEs, this study could be expanded to seek statistical generalizability rather than analytical generalizability, as is the approach of this article. Second, our findings encourage further research into the potential drawbacks of 5G technology. For example, issues involving health risks and new security issues will necessitate additional research in terms of both component elements and dynamics. This study's theoretical arguments could be used to generate several hypotheses for further empirical testing using quantitative research methods.

In practise, the promise of 5G technology's strength of connectivity, high speed, and frequency of data transfers will allow a wide range of highly skilled workers to work from any location or workplace. Similarly, 5G Technology will expand the availability of infrastructure platforms that support electronic marketplaces and promote digital business models. It is also worth noting that supply chains in businesses that have already adopted digitisation stand a better chance of benefiting from 5G technology. Every business, regardless of size or industry, should think about how 5G technology can help them and their customers. However, the features that make 5G so appealing pose new challenges to cyber security. A broader source of concern is that as more devices connect to the network and transmit data through it, new vulnerabilities emerge. Given the prevalence of cloud computing, 5G raises new security concerns. The most effective way to reduce 5G network risk factors is thought to be a zero-trust protection model. Security is based on data verification, validation, and authentication for each access request on a zero-trust basis. While many people will work to maximise the benefits of 5G, others will work to weaken it. Businesses must keep this in mind when implementing 5G or any new technology and align their security policies to stay safe.

## Conclusion

The goal of this article was to establish a link between 5G technology and digital innovation, particularly in SMEs. The article began by establishing the context of the paper by summarising current knowledge and background information on the subject. This was accomplished by stating the purpose of the paper, the research question/problem, briefly explaining the methodological approach, and emphasising the study's potential outcomes. Conceptual framework and synthesis were created to demonstrate the key relationships between the focal constructs (5G technology and digital innovation). This was done to aid comprehension of the conceptual framework. This paper contends that the implementation of 5G technology creates a platform for digital innovation in SMEs' communication, remote working, supply chains, and marketing activities. This can simplify and improve the efficiency of work processes and operations within SMEs, resulting in increased productivity. 5G technology will create enormous opportunities for new revenue streams and will serve as the foundation for business innovation and development on a scale that companies in almost every industry have yet to fully explore.

## Data Availability

Data sharing is not applicable.

## Abbreviations

SMEs: Small and Medium Scale Enterprises
5G: The fifth generation of broadband data-networking digital communications technology
4G: The fourth generation of broadband data-networking digital communications technology
3G: The third generation of broadband data-networking digital communications technology
